# Adhesion forces and mechanics in mannose-mediated acanthamoeba interactions

**DOI:** 10.1371/journal.pone.0176207

**Published:** 2017-05-04

**Authors:** Steven Huth, Julia F. Reverey, Matthias Leippe, Christine Selhuber-Unkel

**Affiliations:** 1 Institute of Materials Science, Biocompatible Nanomaterials, Christian-Albrechts-Universität zu Kiel, Kaiserstr. 2, D-24143 Kiel, Germany; 2 Zoological Institute, Comparative Immunobiology, Christian-Albrechts-Universität zu Kiel, Am Botanischen Garten 1-9, 24118 Kiel, Germany; LAAS-CNRS, FRANCE

## Abstract

The human pathogenic amoeba *Acanthamoeba castellanii* (*A. castellanii*) causes severe diseases, including acanthamoeba keratitis and encephalitis. Pathogenicity arises from the killing of target-cells by an extracellular killing mechanism, where the crucial first step is the formation of a close contact between *A. castellanii* and the target-cell. This process is mediated by the glycocalix of the target-cell and mannose has been identified as key mediator. The aim of the present study was to carry out a detailed biophysical investigation of mannose-mediated adhesion of *A. castellanii* using force spectroscopy on single trophozoites. In detail, we studied the interaction of a mannose-coated cantilever with an *A. castellanii* trophozoite, as mannose is the decisive part of the cellular glycocalix in mediating pathogenicity. We observed a clear increase of the force to initiate cantilever detachment from the trophozoite with increasing contact time. This increase is also associated with an increase in the work of detachment. Furthermore, we also analyzed single rupture events during the detachment process and found that single rupture processes are associated with membrane tether formation, suggesting that the cytoskeleton is not involved in mannose binding events during the first few seconds of contact. Our study provides an experimental and conceptual basis for measuring interactions between pathogens and target-cells at different levels of complexity and as a function of interaction time, thus leading to new insights into the biophysical mechanisms of parasite pathogenicity.

## Introduction

Pathogenic amoebae can cause severe and hard-to-treat infections, ranging from localized infections to life-threatening diseases, such as encephalitis [1]. Still, most amoeba-related diseases occur in regions with limited access to clean water resources. Swimming pools and contact lenses have also been identified as sources of contamination with *Acanthamoeba spp.* and *Naegleria spp.* [2–4].

The genus *Acanthamoeba spp.* consists of species of free-living amoebae [2, 5]. There are both pathogenic acanthamoebae, such as *Acanthamoeba castellanii* [5] or *A. culbertsoni* [6], and non-pathogenic acanthamoebae like *A. comandani* [7]

In recent years, a lot of interest in medical research has focused on *A. castellanii*, as it not only causes severe diseases such as amoebic keratitis and chronic granulomatous amoebic encephalitis [8], but as this thread is further enhanced by the variety of ways for humans to be exposed to them during everyday life. Furthermore, *A. castellanii* can host endosymbionts (e.g. Legionella pneumophila), increasing their pathogenic potential and leading to an increased risk of other infections such as legionnaires’ disease [2, 8]. When brought into the eye, *A. castellanii* invade the corneal epithelium, where they kill cornea epithelial cells in a contact dependent process and cause inflammatory diseases [9–11]. The infection risk is especially high for contact lense users, since acanthamoebae can adhere to the lenses [12] and a growing number of people using contact lenses requires a deeper understanding of basic invasion and cell-killing mechanisms of *A. castellanii* [9, 13].

Although a lot of research has concentrated on *A. castellanii*, most studies focused on improving medication strategies and treatments against acanthamoebae infections. Recent findings implicate an important influence of biophysical factors on the behavior of *A. castellanii*. For example, it has been shown that the cell adhesion area of *A. castellanii* trophozoites depends on substrate stiffness [14]. Another example is that the number of pseudopodia (called acanthopodia [15]) of amoebae trophozoites correlates with their pathogenic potential—non-pathogenic species show less acanthopodia than pathogenic ones. Trogocytosis (internalization of fragments of living cells) [16], cytolysis and ingestion of target cells through food cups are typical cell killing mechanisms of amoebae and the latter two have been already observed in *A. castellanii* [2]. Furthermore, the cell killing mechanism of *A. castellanii* is contact-dependent, meaning that molecular interactions are essential for *A. castellanii* pathogenicity [17].

It has been reported that the presence of the carbohydrate mannose in the membrane of the target cell is necessary for pathogenic acanthamoebae to form contacts to this cells. Indeed, mannose is also part of different adhesion promoting proteins such as laminin and fibronectin [18, 19]. Contact formation of *A. castellanii* is initiated between the mannose on the membrane of target cells and mannose-binding proteins (MBP) in the membrane of the amoebae [20] and subsequently, cytolytic factors move to the contact site and are released leading to target-cell death [17, 19, 21]. Mannose blocking and saturation experiments resulted in reduced adhesion of acanthamoebae to target cells and reduced cytotoxicity and no other molecule has so far shown a similar effect [20, 22, 23]. Furthermore, injuries in the epithelial barrier have been shown to increase the risk of an acanthamoebae infection also for non-contact lens users, which has been connected to a possibly increased mannose content in these areas [24, 25]. A reduced concentration of MBP in non-pathogenic acanthamoebae compared to pathogenic species is another hint pointing at a dependency of pathogenicity on mannose [18]. Hence, a better understanding of mannose-mediated *A. castellanii* interactions could lead to novel strategies for preventing and fighting acanthamoeba-born diseases.

In this study, we measured the interaction forces between mannose and *A. castellanii* using an atomic force microscope (AFM), which is a well-established and highly precise tool to investigate forces from the cellular level down to the level of single ruptures [26]. Using the AFM, we measured the interaction forces of single trophozoites and mannose-coated cantilevers, which mimicked the pathogenicity-relevant part of the glycocalix. We also present a detailed analysis of rupture events that occur during the detachment process. Our data show that mannose is indeed a key factor for the interaction of human pathogenic *A. castellanii* and that its adhesion is independent of the cytoskeleton at the timescales studied here.

## Materials and methods

### Cell culture

*A. castellanii* (ATTC^®^ 30234, derived from ATCC^®^ 30011) and *A. comandoni* (Pb30/40, group 1, genotype T7 [7]) were cultured at room temperature in tissue culture bottles (75 ml, Sarstedt AG & Co., Nümbrecht, Germany). A Peptone Yeast Glucose (PYG) Medium 712 consisting of 20 g proteose peptone (BD, Sparks, USA), 1 g yeast extract (BD, Sparks, USA), 950 ml distilled water, 8 ml 0.05 M CaCl_2_ (AppliChem GmbH, Darmstadt, Germany), 10 ml 0.4 M MgSO_4_ × 7H_2_O (AppliChem GmbH, Darmstadt, Germany), 10 ml 0.25 M Na_2_HPO_4_ × 7H_2_O (Carl Roth GmbH & Co. KG, Karlsruhe, Germany), 10 ml 0.25 M KH_2_PO_4_ (Carl Roth GmbH & Co. KG, Karlsruhe, Germany), 34 ml 0.1 M Na citrate × 2H_2_O (Merck KGaA, Darmstadt, Germany), 10 ml 0.005 M Fe(NH_4_)_2_(SO_4_)_2_ × 6H_2_O (AppliChem GmbH, Darmstadt, Germany), and 50 ml 2 M glucose (Sigma-Aldrich Chemie GmbH, Munich, Germany)) was used. The medium was renewed once a week by shaking the bottles, removing the old medium with swimming acanthamoebae, and adding fresh medium to the remaining cells.

### Adhesion of *A. castellanii* and *A. comandoni* to mannose-agarose and sepharose coated beads

D-mannose agarose beads (Sigma-Aldrich Chemie GmbH, Munich, Germany) and sepharose beads (CL-4B, ø 40 *μ*m to 165 *μ*m, Sigma-Aldrich Chemie GmbH, Munich, Germany) were each diluted in a ratio of 1:10 in PBS and subsequently centrifuged at 1000 rpm for 5 min. The supernatant was removed and the washing procedure was repeated twice. Finally, the cleaned beads were diluted 1:10 in PBS. 3 × 10^5^ *A. castellanii* or *A. comandoni* in 5 ml of PYG medium were seeded into tissue culture bottles (25 ml, Sarstedt AG & Co., Nümbrecht, Germany) and 0.5 ml of one of the bead solutions were added. The samples were observed with an inverted phase contrast microscope (Olympus CKX41) equipped with a camera (C9300, Hamamatsu, Hamamatsu, Japan) and 50 images of each bottle were taken right after the addition, after 2 h and after 4 h incubation time at room temperature. The number of acanthamoebae adhering to the beads and the total number of acanthamoebae in the resulting images were counted.

### Cantilever preparation

The AFM setup used in this study was a CellHesion^®^ 200 AFM head (JPK Instruments, Berlin, Germany) mounted on an IX71 inverted microscope (Olympus Deutschland GmbH, Hamburg, Germany), which was equipped with a colour CCD camera (The Imaging Source Europe GmbH, Bremen, Germany). The instrument was controlled using the CellHesion^®^ 200 Control Software (version 4.3.48, JPK Instruments, Berlin, Germany). Tipless cantilevers (MLCT-O10 or NP-O10, Bruker AFM Probes, Camarillo, USA) were used. Prior to use, each cantilever was calibrated for its spring constant and its sensitivity. Cantilevers were washed for some seconds in acetone (Sigma-Aldrich Chemie GmbH, Munich, Germany), dried in air, washed in double distilled water before being mounted into the AFM setup. The spring constant of a cantilever was calibrated in air using the well-established thermal noise method implemented in the JPK control software. Calibration was carried out three times for each cantilever and the averaged spring constants were used in the analysis. In this study, spring constants ranged from 0.03 N/m up to 0.35 N/m.

After calibration, a cantilever was functionalized with a BSA biotin—streptavidin—*α*-D-mannose-sp-biotin sandwich: A 50 *μ*l drop of BSA biotin (0.5 mg/ml, Sigma-Aldrich Chemie GmbH, Munich, Germany) was put onto a Parafilm^®^ in a six-well plate. The cantilever chip was immersed in the droplet and incubated at room temperature for at least 16 h. After being washed in 1×PBS the procedure was repeated with a 50 *μ*l drop of streptavidin (0.5 mg/ml, Sigma-Aldrich Chemie GmbH, Munich, Germany) for 30 min to 1 h. The cantilever was washed again in 1×PBS before it was immersed into a 50 *μ*l of *α*-D-mannose-sp-biotin (0.1 mg/ml, GlycoTech Corporation, Gaithersburg, Maryland, USA) for 1 h to 2 h. As a final step, the chip was again washed in 1×PBS buffer. Functionalized cantilevers were either used directly or they were stored up to five days in a drop of the carbohydrate-biotin solution in a six well plate sealed with Parafilm^®^ at 5°C.

### Force spectroscopy on *A. castellanii* trophozoites

2 × 10^4^ *A. castellanii* were seeded into a plastic petri dish (Techno Plastic Products AG (TPP), Trasadingen, Switzerland) in PYG medium. After the acanthamoebae adhered, the PYG medium was removed, the cells were washed with 50mM sodium chloride and 1.5 ml of NaCl (50 mM) was added. The petri dish was installed into the PetriDishHeater^™^ (JPK Instruments, Berlin, Germany) and a calibrated and functionalized cantilever was approached to an adhering trophozoite with force setpoints ranging from 8 nN to 16 nN. The effective force applied to the acanthamoebae depends on the migration and adaptation of the acanthamoebae in a single experiment so that the effective contact force is also influenced by the cell. However, as shown in S1 Fig, the measured rupture forces are not significantly influenced by a change of the force setpoint. After a contact time of 0.5 s, 5 s or 10 s respectively, the cantilever was retracted with a speed of 5 *μ*m/s and a force distance curve in constant-height mode was recorded with a sampling rate of 4096 Hz and pulling lengths ranging from 50 *μ*m to 90 *μ*m. For each cell, 20 to 50 curves were recorded and for each contact time, 5 or 6 cells were probed.

### Inhibition of mannose-mediated interactions

To prove the specificity of mannose-mediated binding, control experiments were carried out. *A. castellanii* were incubated for 30 min at room temperature in NaCl containing *α*-D-mannose (50 mM, Sigma-Aldrich Chemie GmbH, Munich, Germany) prior to approaching a mannose functionalized cantilever to a trophozoite. This concentration was chosen according to previous studies [19, 27]. The following force spectroscopy experiments were conducted using 5 s contact time in constant force mode.

### Data processing

The retraction segments of the measured force-distance curves were smoothed, the tilt as well as the y-offset and x-offset were subtracted, and the maximum adhesion force and the work of detachment were calculated using the JPK data processing software spm-5.1.8 (JPK Instruments, Berlin, Germany).

A homemade program written in Matlab^®^ (Mathworks, version R2015a(8.3.0.532)) using a step detection algorithm based on using the 1D edge detector [28] twice was used to detect the bottom and top edges of the last two rupture steps of the retraction segment. Steps smaller than 50 pN were ignored. Furthermore, the program calculated the rupture position (distance from the position of the last force step to the contact point), the length of the tether prior to the last rupture step, the force-loading rate (slope of the force curve prior to the last step), and the single rupture force of the last step. A linear fit was applied to the last 10 *μ*m prior to the last rupture step in order to determine the loading rates. If the distance between the last and the second last rupture step was smaller than 10 *μ*m, the fitting range was changed to this distance to avoid artifacts in the force loading rates (very high positive values) due to fitting through the second last rupture step. Histograms and box plots as well as student’s t-tests for significance were carried out with Origin (OriginLab Coorporation, version 9.1.0 Sr3).

## Results

### Force spectroscopy on single *A. castellanii* trophozoites

**Fig 1:**
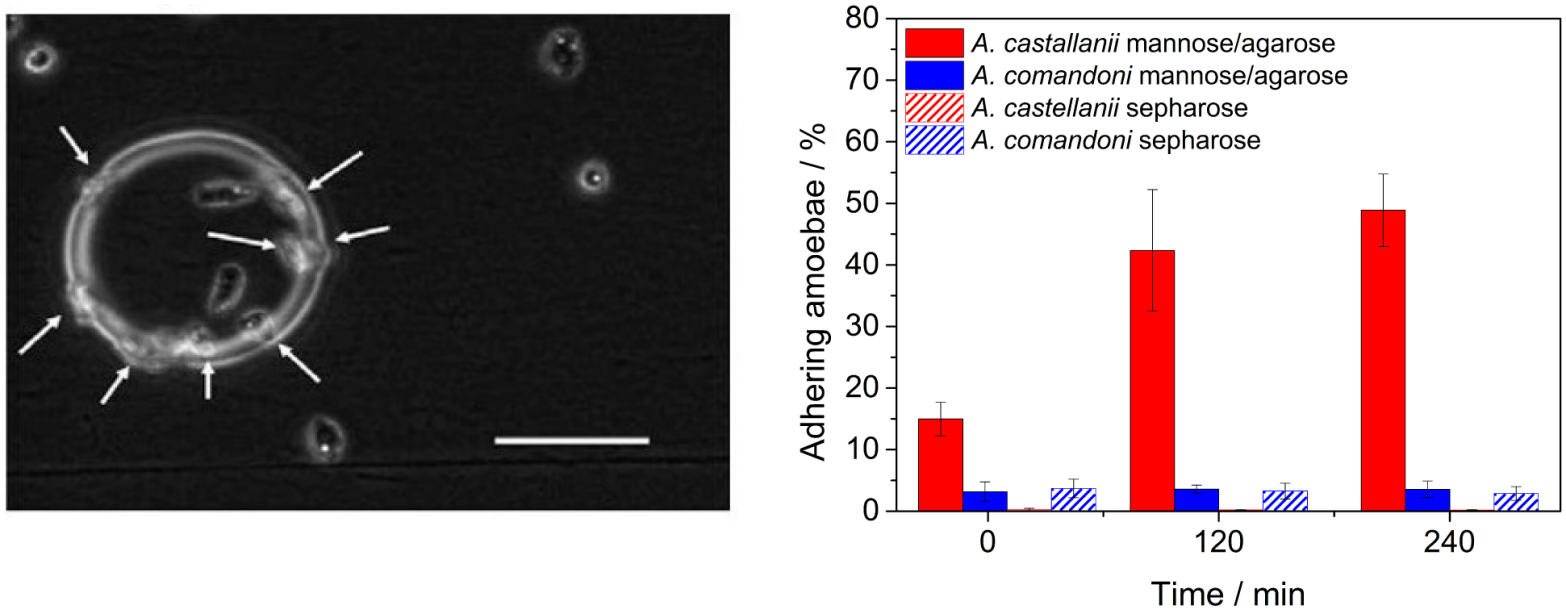
Pathogenic *A. castellanii* adhere to mannose-agarose coated beads. *Left*: *A. castellanii* trophozoites adhering to a mannose-agarose coated bead. White arrows indicate trophozoites at the edge of the sphere. The amount of acanthamoebae adhering to the bead and the total amount of acanthamoebae were counted directly, 2 h and 4 h after cell seeding. *Right*: Comparison of *A. castellanii* and *A. comandoni* adhesion to mannose-agarose and sepharose beads. Only a few *A. comandoni* adhered to the sepharose or mannose-agarose coated beads. A large amount of *A. castellanii* adhered already at short times and the number of adhering acanthamoeba strongly increased with time. Only a few *A. castellanii* adhered to sepharose beads. Scalebar: 100*μ*m.

To confirm that exclusively pathogenic species of acanthamoebae form adhesive contacts in a mannose dependent manner, we cultured *A. castellanii* and *A. comandoni* in proximity of mannose-agarose or sepharose coated beads. The amount of amoebae adhering to the beads as well as the total number of amoebae were counted directly, 2h and 4h after cell seeding. The results and a representative image of the experiment are presented in Fig 1. The *A. castellanii* trophozoites adhere exclusively to mannose-agarose coated beads and the number of adhering acanthamoebae increases with time, but only for *A. castellanii*. No significant adhesion of *A. castellanii* to sepharose beads was observed. Only very few *A. comandoni* adhered to both mannose-agarose and sepharose beads. No difference could be observed in the adhesive interaction of *A. comandoni* with manose-agarose or sepharose beads. Due to these differences, and as only pathogenic *A. castellanii* displayed adhesion to the mannose-agarose beads we conducted a further, more detailed study of the mannose dependent adhesive behavior of *A. castellanii* using AFM based force spectroscopy to learn more about the binding dynamics of these parasites to mannose.

**Fig 2:**
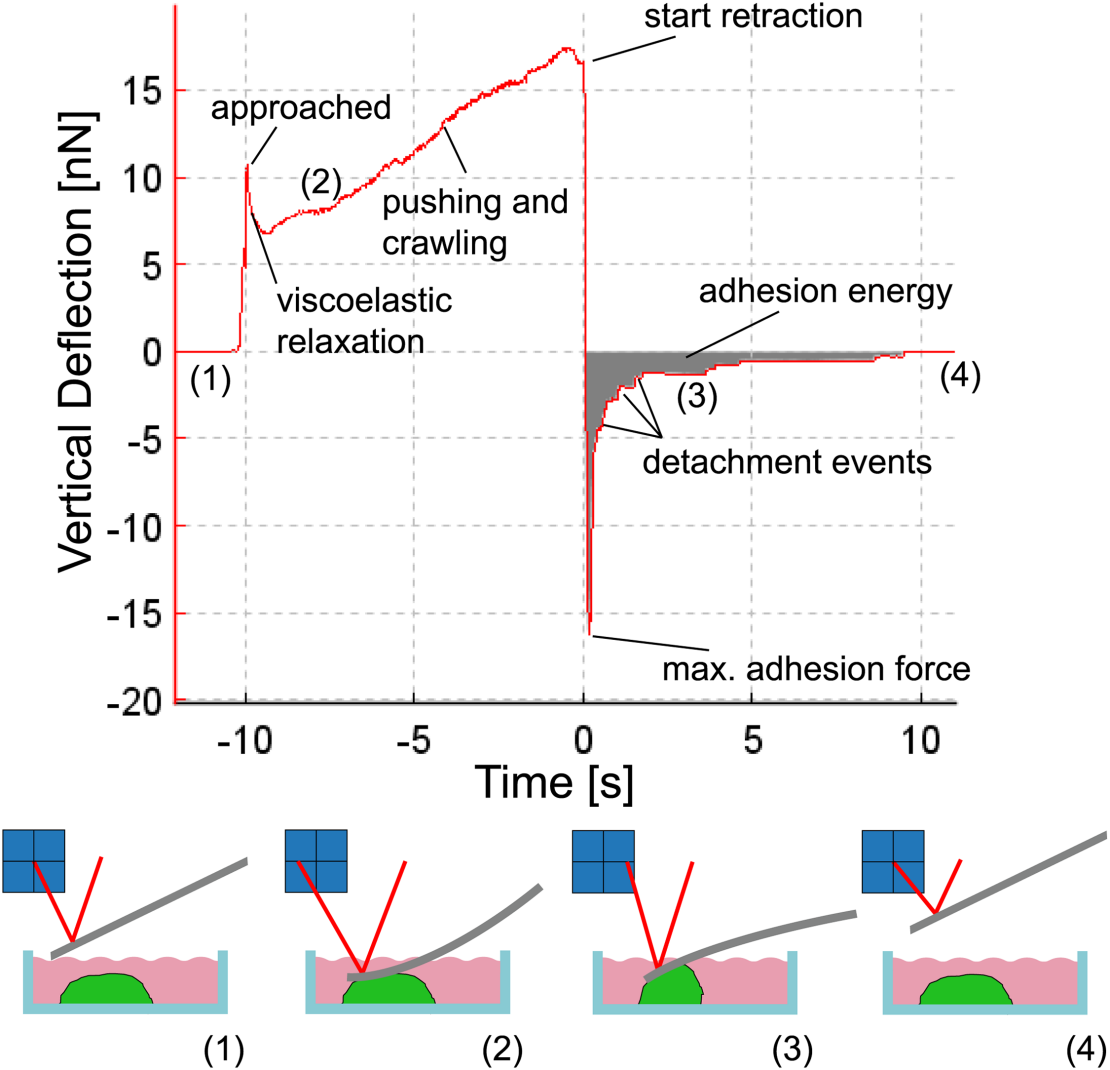
Force versus time curve resulting from an AFM measurement on an *A. castellanii*. The contact time was 10 s. The cantilever is approached to the cell at -10 s and the force increases to the setpoint value before it decreases due to viscoelastic relaxation. Subsequently the force increases again to values much larger than the setpoint as the acanthamoeba pushes against the cantilever. At 0 s the cantilever is retracted and detachement of cellular adhesions can be observed as steps in the force data.

Fig 2 presents an example of a force-distance curve while probing the adhesion of an *A. castellanii* trophozoite with the AFM. The cantilever is approached to the cell (1) until a set-point force is reached (“approached”). During contact (dwell time), the cell has time to viscoelastically relax and also to form new contacts to the cantilever, which is bent due to the active pushing and crawling of the amoeba (2). After a defined contact time, the retraction starts and the cantilever is bent into the opposite direction as the cell withstands the pulling due to its adhesion to the surface of the petri dish. After a certain force (the so-called maximum adhesion force) is reached, the contacts between the cell and the cantilever rupture and a step like pattern can be observed in the force curve (3). The height of one step shows the force that was necessary to break the connections between cell and cantilever and the length and slope of the horizontal part of the step gives information on loading rate (dF/ds) and the nature of the ruptured contact. Contacts between the cantilever and the cytoskeleton are mechanically very stable and rigid and show a strongly negative slope before the rupture event while slopes close to zero indicate membrane tethering. A tether is a tubular structure that is pulled out of the cellular membrane if the cantilever is exclusively connected to this membrane [29, 30]. After all contacts are ruptured, the cantilever is no longer in contact with the cell and no further cantilever bending is observed (4). Cantilever deflections are converted into forces using a calibration procedure prior to an experiment.

The data that can be gained from such force-distance curves include the total detachment force (the force necessary to initiate the cantilever detachment from the cell) denoted by the minimum of the force curve, the work of detachment (work required to fully detach cantilever from the cell), which is the area under the force curve, the last rupture force (height of the last rupture event, describing the final connection between cantilever and cell), the slope prior to the last rupture event (characterizing the mechanical connection between cantilever and cell) and, in case of tether events, the length of the last tether, as this provides a force clamp to the final connection between cell and cantilever [29]. For single rupture investigations, only the last rupture event is evaluated as earlier events may present artifacts in single rupture forces as several molecules may be ruptured simultaneously or as other existing adhesions can be loaded with forces and hence lower the rupture force [26].

**Fig 3:**
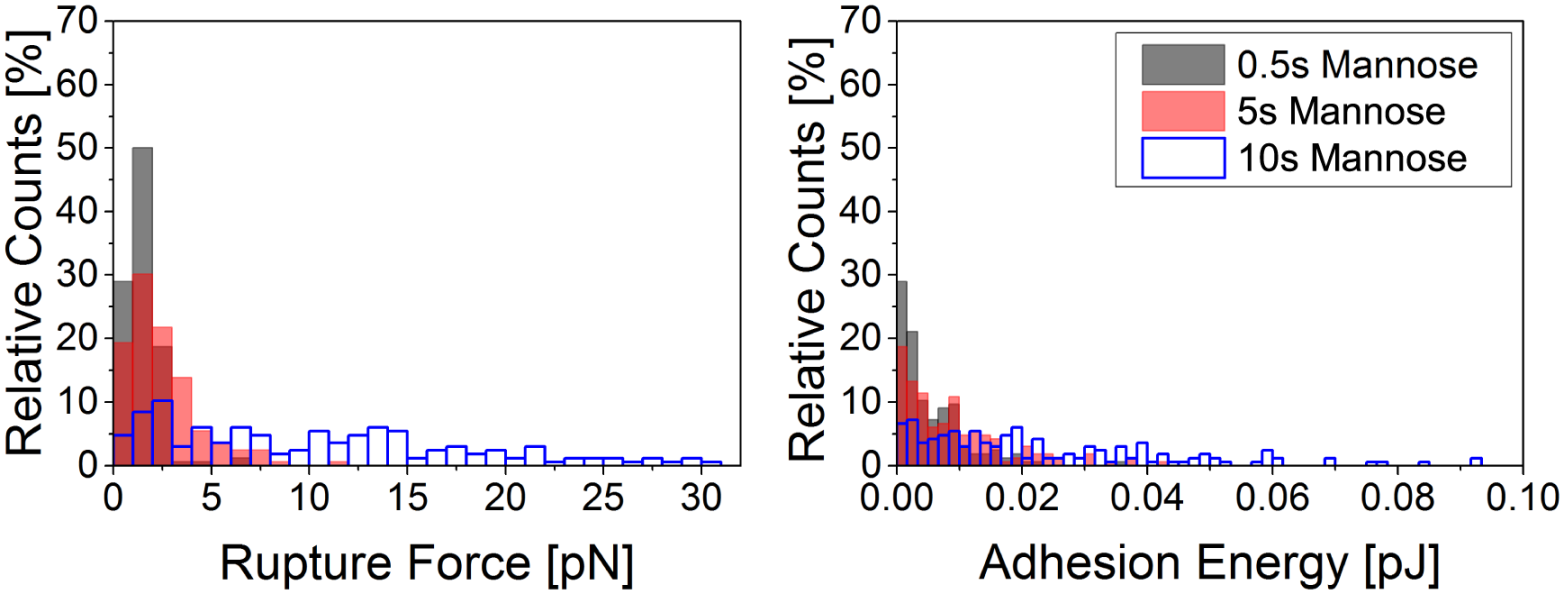
Histogram of total rupture forces (left) and work of detachment (right) between a mannose coated AFM cantilever and an *A. castellanii* trophozoite after 0.5 s, 5 s and 10 s contact time, respectively. Adhesion forces and work of detachment increase with time. Comparably high forces between 0.5 nN and 30 nN were measured.

For this study, we approached a mannose functionalized cantilever to an isolated adhering *A. castellanii* trophozoite. After contact times of 0.5 s, 5 s or 10 s, the cantilever was retracted and force-distance curves were recorded. For each contact time, several cells were probed and many force curves were recorded per cell. First, we evaluated if different contact times lead to differences in the total rupture forces and work of detachment. The results are presented in Fig 3 and clearly show that longer contact times lead to higher total rupture forces as well as higher work of detachment. Very high total rupture forces of up to 30 nN were recorded. We assume that the cantilever coating was not changed throughout the recording of the force curves (see S2 Fig).

**Fig 4:**
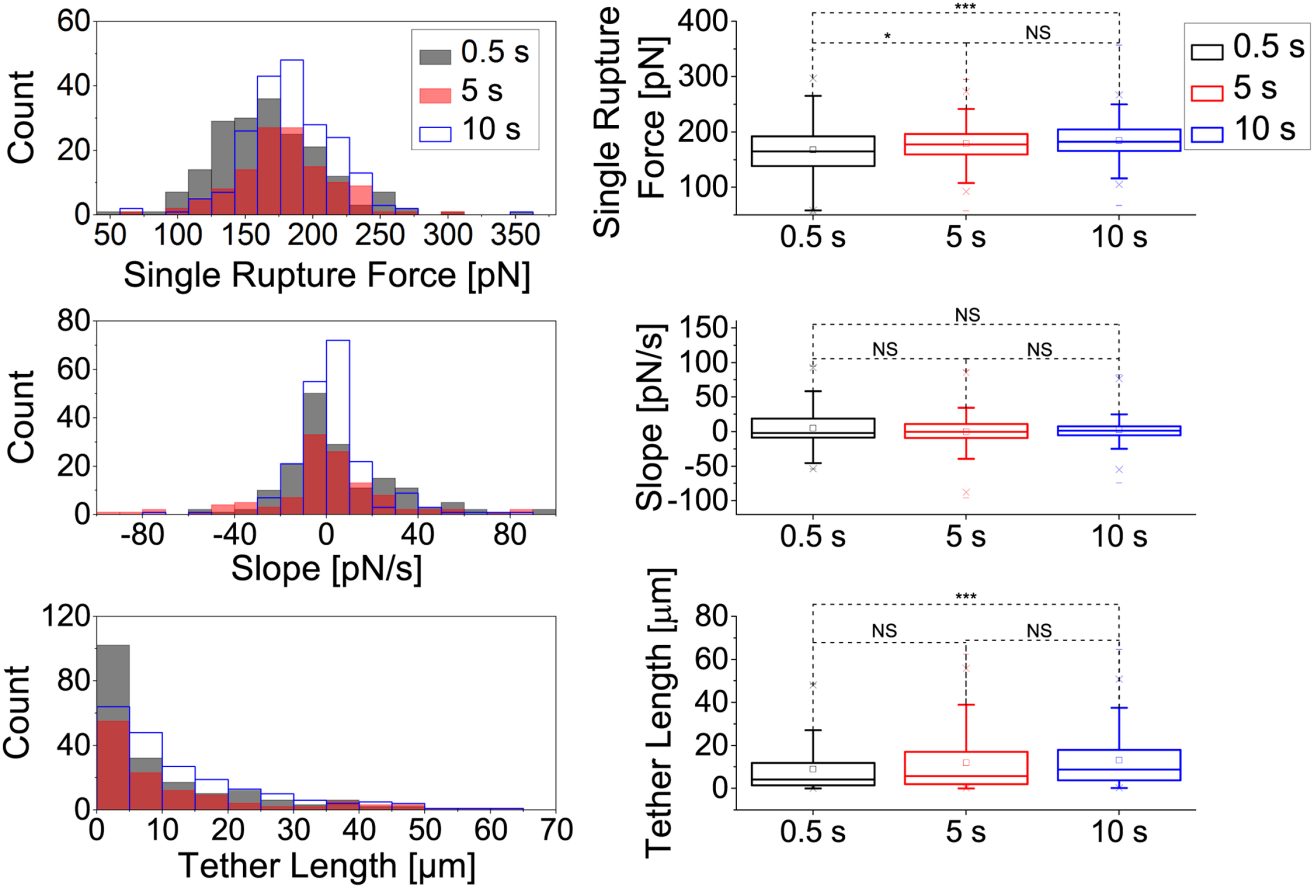
Single rupture analysis of the last rupture event between a mannose-coated AFM cantilever and an *A. castellanii* cell after 0.5 s, 5 s and 10 s contact time. Histograms (left) and boxplots (right) of rupture forces (top), slopes (center) and tether lenghts (bottom) of the last rupture event. Significant changes in rupture forces were observed between 0 s and 5 s (*, p≤0.05), and 0 s and 10 s (***, p≤0.001) contact time. The slopes were not significantly changed by the duration of cantilever-amoeba contact. All measured slopes were above -100 pN/s. Significant changes of tether lengths were only recorded between 0 s and 10 s contact time (***, p≤0.001).

We intended to further understand the influence of time on cell adhesion rupture forces by investigating how single rupture events depend on contact time. We examined the force necessary to induce the last rupture event of each force curve as well as the slope and length of the tether prior to this rupture event. The results are shown in Fig 4. A weakly significant difference (p≤0.05) of last single rupture forces was recorded between 0 s and 5 s contact time and a strongly significant difference (p≤0.001) between 0 s and 10 s contact time. No significant change of last single rupture forces was recorded between 5 s and 10 s contact time suggesting that no huge adhesion clusters are formed. Still, the formation of tiny clusters cannot fully be excluded due to the statistically significant increase in single rupture force between 0.5 and 10 sec contact time. The slopes prior to the rupture event were not significantly affected by the duration of cell cantilever contact and all measured slopes were above -100 pN/s. A significant change in tether lengths was only recorded between 0 s and 10 s contact time (p≤0.001)

**Fig 5:**
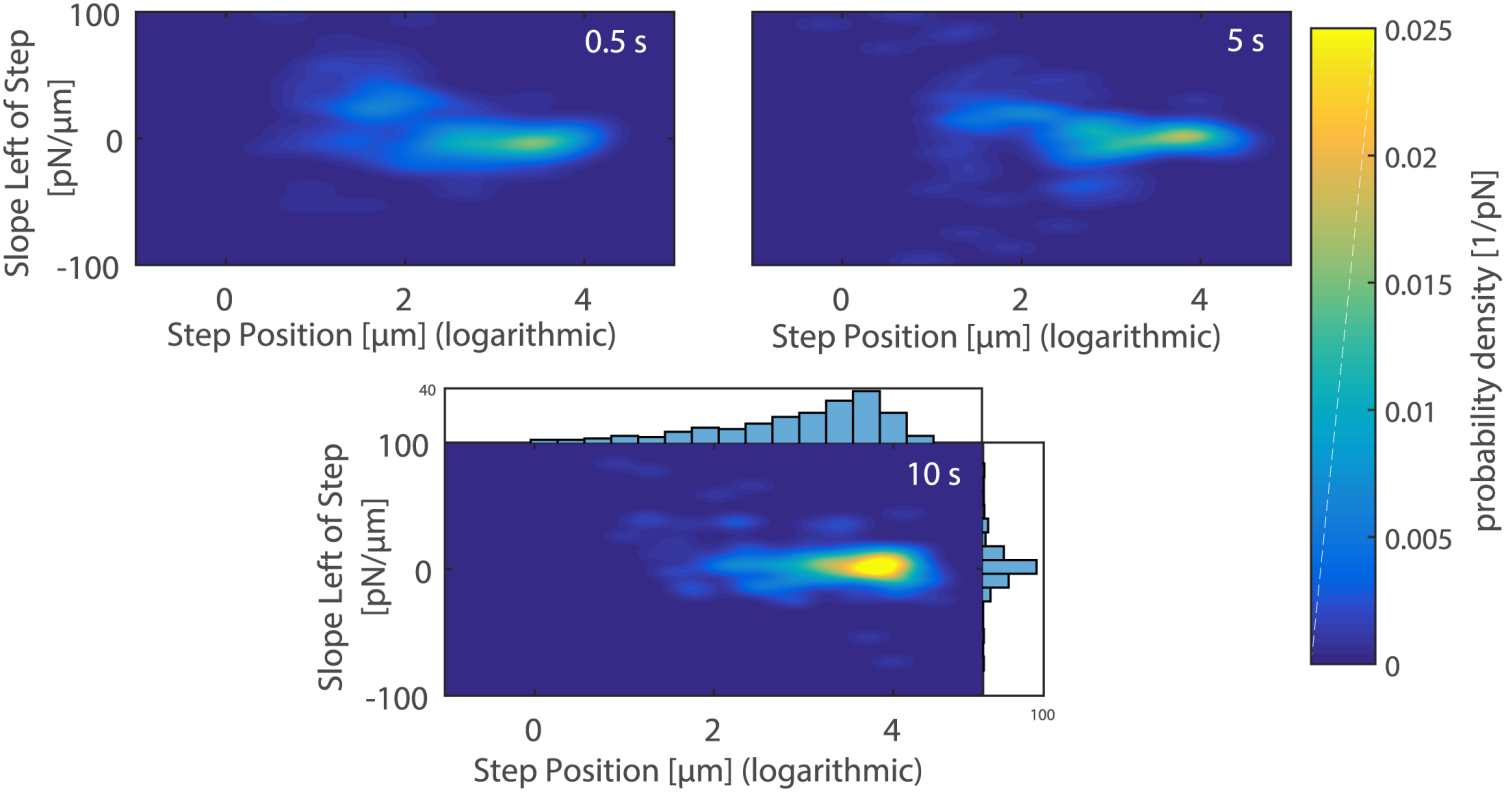
2D probability density plots of slopes versus position of the last rupture event when breaking the contact between a mannose coated AFM cantilever and a *A. castellanii* cell after 0.5 s (top-left), 5 s (top-right) and 10 s (bottom) contact time. Higher contact times result in higher probability densities at higher rupture positions and narrower slope distributions. Sariisik et al. stated that slopes above -30 pN/*μ*m indicate tethering prior to the rupture event [31]. Since our cantilever retraction speed was 5 *μ*m, tethers occur if slopes are above -150 pN/s, which is the case for all our measurements. The figure on the bottom also includes the corresponding histograms for slope and rupture position distributions. The axes of ordinates of these histograms represent counts.

Fig 5 presents the two-dimensional probability density plots of slopes versus position of the last rupture event. Information on the mechanical nature of the bonds between the *A. castellanii* trophozoite and the mannose coated cantilever can be drawn out of these plots. Higher probability densities at higher rupture positions and narrower slope distributions were measured for higher contact times.

**Fig 6:**
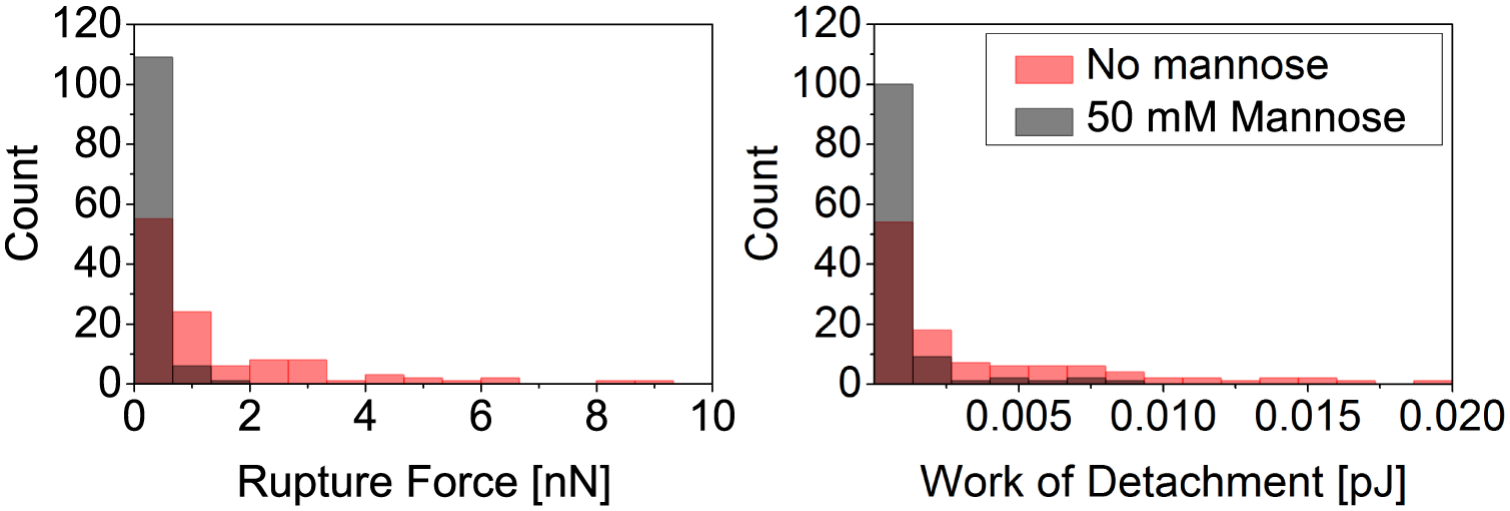
Control experiments using mannose in the medium to prove the specificity of binding. The data show that *A. castellanii* trophozoite interaction with the mannose-coated cantilever is specific. Histogram of total rupture forces (left) and work of detachment (right) for the contact between a mannose coated AFM cantilever and a *A. castellanii* trophozoite after 5 s contact time. Cells that have been incubated in medium containing mannose prior to the experiment show much weaker adhesion than cells in medium without mannose.

To show that the contacts formed between cantilevers and *A. castellanii* trophozoites were based on specific bonds between mannose and MBP, we performed control experiments where mannose receptors were blocked by exposing cells to a high concentration of mannose in the medium. In Fig 6 the total rupture forces and work of detachment needed to detach a mannose functionalized cantilever from *A. castellanii* incubated in mannose and non-incubated *A. castellanii* trophozoites are presented. Clearly, incubating the trophozoites in mannose prior to the force spectroscopy experiments leads to much lower rupture forces and work of detachment.

## Discussion

Previous studies on the mannose-mediated adhesion of acanthamoebae have shown that their pathogenicity is related to the expression of an MBP and therefore to adhesion to mannose [17–19]. The aim of our study was therefore to gain a detailed biophysical insight into adhesion and binding mechanics during the contact-mediated target-cell killing process of acanthamoeba by investigating mannose-mediated binding events. As a first result, we found a clearly increased adhesion of human pathogenic *A. castellanii* to mannose-agarose beads compared to non-pathogenic *A. comandoni*, which might result from a higher expression of MBPs in pathogenic *A. castellanii*.

Due to this finding a particular focus of our study was the investigation of mannose-mediated binding of *A. castellanii* trophozoites down to the level of single rupture events using force spectroscopy on single trophozoites. It is noteworthy that force spectroscopy on acanthamoebae requires a few modifications to the classical Single-Cell Force Spectroscopy experiment used for mammalian cells. There, the cell is typically glued to the cantilever [26, 32] and contact times of several minutes can easily be probed with the cell as an almost spherical object at the cantilever [33]. This is not possible for acanthamoebae due to their strong motility, which pushes the cantilever up and pulls it down. Instead, we needed to use the cantilever as a mannose-coated probe (to mimic the contact-relevant parts of the coat of the target-cell) and chose a relatively high force setpoint to hinder the acanthamoeba from crawling away from the cantilever. The magnitude of total detachment force measured in our experiments is striking, as interaction forces of up to 30 nN were observed. This high force might be caused by the large contact area between the tipless cantilever and the trophozoite, which was induced by a high setpoint in contact force between cantilever and cell.

In general, we observed a strong dependency of forces and work necessary to detach a mannose-coated cantilever from an *A.castellanii* trophozoite on contact time (Fig 3, which is typical for adhesive cells [33]). Mannose binding turned out to be a relatively fast process as detachment forces of up to 7 nN were measured for 0.5 s contact time, whereas we measured forces of 150 pN to 200 pN for single ruptures.

By evaluating the slopes prior to the last rupture event we found that the slopes were above -100 pN/s for all investigated contact times. Sariisik et al. stated that slopes above -30 pN/*μ*m indicate the pulling of tether from the trophozoite membrane prior to the rupture event. Considering our pulling speed of 5 *μ*m/s this value becomes -150 pN/s. A tether is a thin membrane tube that is often pulled out from the cell membrane when a force is applied to a membrane-bound receptor, which is not connected to the cytoskeleton. This prevents the receptor from being loaded with further force and the tether acts as a force clamp. In such cases the receptor-ligand bond does not rupture due to an increase in external load, but rather because the force clamp is applied for an extended time and thus increases the probability of the bond to overcome the energy barrier to the unbound state [29, 30]. Recently, Sariisik et al. have shown that the cytoskeletal coupling of bonds measured with force spectroscopy on single cells can be studied using two-dimensional probability density plots of rupture position versus slope [31]. Using this representation on our data, we could show that exclusively tethers without cytoskeletal coupling are associated with the last rupture events for all studied contact times. This is very interesting, as acanthamoeba migration and adhesion to surfaces is indeed strongly influenced by the cytoskeleton and molecular motors [34, 35], whereas mannose-mediated binding is according to our data independent of the cytoskeleton. As mannose-mediated binding in pathogenic killing events relies on short-term interactions between *A. castellanii* and target-cells, we therefore assume that for this very specific and short-term interaction the cytoskeleton plays only a minor role. In addition, analysis of the magnitude of single rupture events has shown that their average values only increased slightly with contact time. This indicates that mannose-mediated interactions rather rely on single molecule binding or on smaller clusters than on larger adhesion clusters, which could be an explanation for the high pathogenicity and thus efficient target-cell killing of acanthamoeba.

In summary, we present here for the first time data of single rupture event interactions between pathogenic *A. castellanii* and a mannose-coated cantilever, which mimicks that part of the target-cell’s glycocalix that is relevant for the cytolytic reaction and thus for target-cell killing. Large forces and energies are needed to detach an *A. castellanii* completely from the cantilevers and these increase with contact time, which is reasonable as more and more mannose binding proteins can diffuse into the contact region with time. However, on a single rupture level, the rupture forces increased only slightly, indicating that adhesion strengthening by the formation of large adhesion clusters can be excluded. The acanthamoebae apparently form very quickly very strong bonds between the MBP proteins in their membranes and the mannose on target cells. These bonds are not stably connected to the acanthamoeba’s cytoskeleton, as our experiments have exclusively shown anchorage of MBP in the membrane. Our study can serve as a starting point for future force spectroscopic studies on cellular interactions that are related to receptor-mediated, pathogenic reactions in parasites.

## Supporting information

S1 Fig Different force setpoints did not significantly change the measured rupture forces. The measured rupture forces are plotted versus the number of measurement. After force curve 80 (marked with the horizontal line) the cantilever was changed. This lead to a change of the force setpoint from 19 nN to 11 nN. However, a change of measured rupture forces was not observed.
(PDF)

S2 Fig Mannose functionalization of the cantilever does not alter with time. The measured rupture forces are plotted versus the number of force curves that already were recorded with the utilized cantilever. 56 curves, which were recorded on one cell with 10 s contact time, were taken into account. This is the maximum amount of time a cantilever has been used on one cell during this study. The data do not show a decline of rupture forces with time for these 56 curves.
(PDF)

## References

[1] Martinez AJ, Visvesvara GS. Free-living, Amphizoic and Opportunistic Amebas. Brain Pathology. 1997 1;7(1):583–598. Available from: http://doi.wiley.com/10.1111/j.1750-3639.1997.tb01076.x doi: 10.1111/j.1750-3639.1997.tb01076.x 9034567 10.1111/j.1750-3639.1997.tb01076.x PMC8098488

[2] Marciano-Cabral F, Cabral G. Acanthamoeba spp. as agents of disease in humans. Clinical Microbiology Reviews. 2003;16(2):273–307. doi: 10.1128/CMR.16.2.273-307.2003 12692099 10.1128/CMR.16.2.273-307.2003 PMC153146

[3] Ithoi I, Ahmad AF, Nissapatorn V, Lau YL, Mahmud R, Mak JW. Detection of Naegleria species in environmental samples from peninsular Malaysia. PLoS ONE. 2011;6(9):1–10. doi: 10.1371/journal.pone.0024327 10.1371/journal.pone.0024327 PMC3167841 21915311

[4] Caumo K, Rott MB. Acanthamoeba T3, T4 and T5 in swimming-pool waters from Southern Brazil; 2011. 10.1016/j.actatropica.2010.12.008 21195045

[5] Khan NA. Acanthamoeba: Biology and Pathogenesis. Caister Academic Press; 2009.

[6] Michalek M, Sönnichsen FD, Wechselberger R, Dingley AJ, Hung CW, Kopp A, et al Structure and function of a unique pore-forming protein from a pathogenic acanthamoeba. Nat Chem Biol. 2013 1;9(1):37–42. Available from: http://dx.doi.org/10.1038/nchembio.1116http://www.nature.com/nchembio/journal/v9/n1/abs/nchembio.1116.html#supplementary-information doi: 10.1038/nchembio.1116 23143413 10.1038/nchembio.1116

[7] Michel R, Steinert M, Zöller L, Hauröder B, Henning K. Free-living Amoebae May Serve as Hosts for the Chlamydia-like Bacterium Waddlia chondrophila Isolated from an Aborted Bovine Foetus. Acta Protozoologica. 2004;43(1):37–42.

[8] Khan NA. Acanthamoeba: Biology and increasing importance in human health. FEMS Microbiology Reviews. 2006;30(4):564–595. doi: 10.1111/j.1574-6976.2006.00023.x 16774587 10.1111/j.1574-6976.2006.00023.x

[9] Larkin DFP, Kilvington S, Easty DL. Contamination of contact lens storage cases by Acanthamoeba and bacteria. The British Journal of Ophthalmology. 1990;74:133–135. Available from: http://www.pubmedcentral.nih.gov/articlerender.fcgi?artid=1042032&tool=pmcentrez&rendertype=abstract doi: 10.1136/bjo.74.3.133 2322508 10.1136/bjo.74.3.133 PMC1042032

[10] Lorenzo-Morales J, Martín-Navarro CM, López-Arencibia A, Arnalich-Montiel F, Piñero JE, Valladares B. Acanthamoeba keratitis: an emerging disease gathering importance worldwide? Trends in Parasitology. 2013;29(4):181–187. doi: 10.1016/j.pt.2013.01.006 23433689 10.1016/j.pt.2013.01.006

[11] Moore MB, McCulley JP, Newton C, Cobo LM, Foulks GN, O’Day DM, et al Acanthamoeba Keratitis: A Growing Problem in Soft and Hard Contact Lens Wearers. Ophthalmology. 1987;94(12):1654–1661. doi: 10.1016/S0161-6420(87)33238-5 3431835

[12] Reverey JF, Fromme R, Leippe M, Selhuber-Unkel C. In vitro adhesion of Acanthamoeba castellanii to soft contact lenses depends on water content and disinfection procedure. Contact lens & anterior eye: the journal of the British Contact Lens Association. 2014 8;37(4):262–6. Available from: http://www.contactlensjournal.com/article/S1367048413003159/fulltext doi: 10.1016/j.clae.2013.11.010 24361096 10.1016/j.clae.2013.11.010

[13] Dart JKG, Stapleton F, Minassian D, Dart JKG. Contact lenses and other risk factors in microbial keratitis. The Lancet. 1991;338(8768):650–653. doi: 10.1016/0140-6736(91)91231-I 10.1016/0140-6736(91)91231-i 1679472

[14] Gutekunst SB, Grabosch C, Kovalev A, Gorb SN, Selhuber-Unkel C. Influence of the PDMS substrate stiffness on the adhesion of Acanthamoeba castellanii. Beilstein Journal of Nanotechnology. 2014;5(1):1393–1398. doi: 10.3762/bjnano.5.152 25247122 10.3762/bjnano.5.152 PMC4168941

[15] Niederkorn JY, Alizadeh H, Leher H, McCulley JP. The pathogenesis of Acanthamoeba keratitis. Microbes and Infection. 1999;1(6):437–443. doi: 10.1016/S1286-4579(99)80047-1 10602676 10.1016/s1286-4579(99)80047-1

[16] Ralston KS, Solga MD, Mackey-Lawrence NM, Somlata, Bhattacharya A, Petri WA Jr. Trogocytosis by Entamoeba histolytica contributes to cell killing and tissue invasion. Nature. 2014 4;508(7497):526–530. Available from: http://dx.doi.org/10.1038/nature13242http://10.0.4.14/nature13242http://www.nature.com/nature/journal/v508/n7497/abs/nature13242.html#supplementary-information doi: 10.1038/nature13242 24717428 10.1038/nature13242 PMC4006097

[17] Garate M, Cao Z, Bateman E, Panjwani N. Cloning and characterization of a novel mannose-binding protein of Acanthamoeba. Journal of Biological Chemistry. 2004;279(28):29849–29856. doi: 10.1074/jbc.M402334200 15117936 10.1074/jbc.M402334200

[18] Garate M, Marchant J, Cubillos I, Cao Z, Khan NA, Panjwani N. In vitro pathogenicity of Acanthamoeba is associated with the expression of the mannose-binding protein. Investigative Ophthalmology and Visual Science. 2006;47(3):1056–1062. doi: 10.1167/iovs.05-0477 16505041 10.1167/iovs.05-0477

[19] Cao Z, Jefferson DM, Panjwani N. Role of carbohydrate-mediated adherence in cytopathogenic mechanisms of Acanthamoeba. Journal of Biological Chemistry. 1998;273(25):15838–15845. doi: 10.1074/jbc.273.25.15838 9624184 10.1074/jbc.273.25.15838

[20] Yang Z, Panjwani N. Pathogenesis of acanthamoeba keratitis: Carbohydrate-mediated host-parasite interactions. Investigative Ophthalmology and Visual Science. 1997;38(4):439–445. 10.1128/iai.65.2.439-445.1997 PMC174614 9009294

[21] Panjwani N. Pathogenesis of Acanthamoeba Keratitis. The Ocular Surface. 2010;8(2):70–79. doi: 10.1016/S1542-0124(12)70071-X 20427010 10.1016/s1542-0124(12)70071-x PMC3072032

[22] Leher H, Silvany R, Alizadeh H, Huang J, Niederkorn JY. Mannose induces the release of cytopathic factors from Acanthamoeba castellanii. Infection and Immunity. 1998;66(1):5–10. 9423832 10.1128/iai.66.1.5-10.1998 PMC107851

[23] Kim JH, Matin A, Shin HJ, Park H, Yoo KT, Yuan XZ, et al Functional roles of mannose-binding protein in the adhesion, cytotoxicity and phagocytosis of Acanthamoeba castellanii. Experimental Parasitology. 2012;132(2):287–292. doi: 10.1016/j.exppara.2012.08.007 22940016 10.1016/j.exppara.2012.08.007

[24] Sharma S, Garg P, Rao GN. Patient characteristics, diagnosis, and treatment of non-contact lens related Acanthamoeba keratitis. The British journal of ophthalmology. 2000;84(10):1103–8. doi: 10.1136/bjo.84.10.1103 11004092 10.1136/bjo.84.10.1103 PMC1723254

[25] Klink F, Alizadeh H, He YG, Mellon JA, Silvany RE, McCulley JP, et al The role of contact lens. trauma and Langerhans Cell in a Chinese Hamster Model of Acanthamoeba Keratitis. Invest Ophthalmol Vis Sci. 1993;34(6):1937–1944. 8491547

[26] Li Q, Huth S, Adam D, Selhuber-Unkel C. Reinforcement of integrin-mediated T-Lymphocyte adhesion by TNF-induced Inside-out Signaling. Scientific reports. 2016;6(July):30452 Available from: http://dx.doi.org/10.1038/srep30452%0Ahttp://www.ncbi.nlm.nih.gov/pubmed/27466027%0Ahttp://www.pubmedcentral.nih.gov/articlerender.fcgi?artid=PMC4964354 doi: 10.1038/srep30452 27466027 10.1038/srep30452 PMC4964354

[27] Gordon VR, Asem EK, Vodkin MH, McLaughlin GL. Acanthamoeba binds to extracellular matrix proteins in vitro. Investigative ophthalmology & visual science. 1993;34(3):658–662. 8449684

[28] http://www.cs.unc.edu/nanowork/cismm/download/edgedetector/index.html; 2016 CISMM at UNC-CH, supported by the NIH NIBIB (NIH 5-P41-RR02170).

[29] Friedrichs J, Legate KR, Schubert R, Bharadwaj M, Werner C, Müller DJ, et al A practical guide to quantify cell adhesion using single-cell force spectroscopy. Methods (San Diego, Calif). 2013 4;60(2):169–178. Available from: http://www.ncbi.nlm.nih.gov/pubmed/23396062 doi: 10.1016/j.ymeth.2013.01.006 10.1016/j.ymeth.2013.01.006 23396062

[30] Benoit M, Selhuber-Unkel C. Measuring Cell Adhesion Forces: Theory and Principles In: Braga PC, Ricci D, editors. Atomic Force Microscopy in Biomedical Research. Humana Press; 2011 p. 355–377. 10.1007/978-1-61779-105-5_21 21660737

[31] Sariisik E, Popov C, Müller JP, Docheva D, Clausen-Schaumann H, Benoit M. Decoding Cytoskeleton-Anchored and Non-Anchored Receptors from Single-Cell Adhesion Force Data. Biophysical Journal. 2015;109(7):1330–1333. doi: 10.1016/j.bpj.2015.07.048 26445433 10.1016/j.bpj.2015.07.048 PMC4601042

[32] Kadem LF, Holz M, Suana KG, Li Q, Lamprecht C, Herges R, et al Rapid Reversible Photoswitching of Integrin-Mediated Adhesion at the Single-Cell Level. Advanced Materials. 2016;28(9):1799–1802. doi: 10.1002/adma.201504394 26685922 10.1002/adma.201504394

[33] Selhuber-Unkel C, López-García M, Kessler H, Spatz JP. Cooperativity in Adhesion Cluster Formation during Initial Cell Adhesion. Biophysical Journal. 2008;95(11):5424–5431. doi: 10.1529/biophysj.108.139584 18689459 10.1529/biophysj.108.139584 PMC2586591

[34] Reverey JF, Jeon JH, Bao H, Leippe M, Metzler R, Selhuber-Unkel C. Superdiffusion dominates intracellular particle motion in the supercrowded cytoplasm of pathogenic Acanthamoeba castellanii. Scientific Reports. 2015;5(February):11690 Available from: http://www.nature.com/doifinder/10.1038/srep11690 doi: 10.1038/srep11690 26123798 10.1038/srep11690 PMC5155589

[35] Dufour AC, Olivo-Marin JC, Guillen N. Amoeboid movement in protozoan pathogens. Seminars in Cell & Developmental Biology. 2015;46:128–134. doi: 10.1016/j.semcdb.2015.10.010 26459974 10.1016/j.semcdb.2015.10.010

