# Supplementary material for: Adhesion forces and mechanics in mannose-mediated acanthamoeba interactions
Source: PLoS One. 2017 May 4;12(5):e0176207. doi: 10.1371/journal.pone.0176207 (PMC5417443; doi:10.1371/journal.pone.0176207)
Supplement: S1 Fig — The measured rupture forces are plotted versus the number of measurement. After force curve 80 (marked with the horizontal line) the cantilever was changed. This lead to a change of the force setpoint from 19 nN to 11 nN. However, a change of measured rupture forces was not observed. (PDF) [file pone.0176207.s001.pdf]

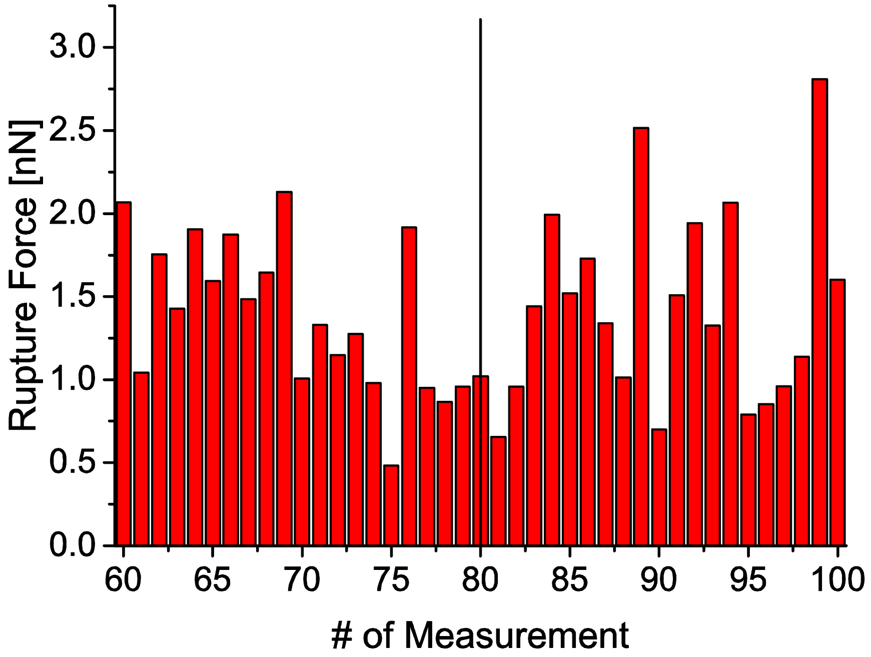


**Figure S1: Different force setpoints did not significantly change the measured rupture forces.** The measured rupture forces are plotted versus the number of measurement. After force curve 80 (marked with the horizontal line) the cantilever was changed. This lead to a change of the force setpoint from 19 nN to 11 nN. However, a change of measured rupture forces was not observed.
