# Supplementary material for: Adhesion forces and mechanics in mannose-mediated acanthamoeba interactions
Source: PLoS One. 2017 May 4;12(5):e0176207. doi: 10.1371/journal.pone.0176207 (PMC5417443; doi:10.1371/journal.pone.0176207)
Supplement: S2 Fig — The measured rupture forces are plotted versus the number of force curves that already were recorded with the utilized cantilever. 56 curves, which were recorded on one cell with 10 s contact time, were taken into account. This is the maximum amount of time a cantilever has been used on one cell during this study. The data do not show a decline of rupture forces with time for these 56 curves. (PDF) [file pone.0176207.s002.pdf]

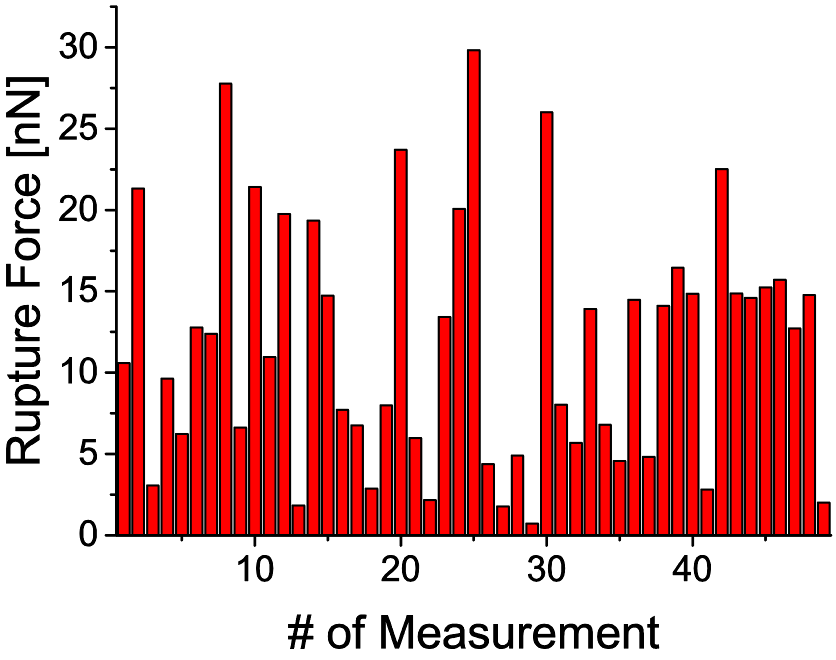

Figure S2: Mannose functionalization of the cantilever does not alter with time. The measured rupture forces are plotted versus the number of force curves that already were recorded with the utilized cantilever. 56 curves, which were recorded on one cell with 10 s contact time, were taken into account. This is the maximum amount of time a cantilever has been used on one cell during this study. The data do not show a decline of rupture forces with time for these 56 curves.
